# Predicting of Process Parameters for Theoretical Concentrated Stress of Fatigue Notch Coefficient of Auto Parts Using Virtual Recognizable Performance Evaluation Research

**DOI:** 10.3390/polym14153043

**Published:** 2022-07-27

**Authors:** Hanjui Chang, Shuzhou Lu, Yue Sun, Guangyi Zhang

**Affiliations:** 1 Department of Mechanical Engineering, College of Engineering, Shantou University, Shantou 515063, China; 21szlu@stu.edu.cn (S.L.); suny09222022@163.com (Y.S.); 20gyzhang1@stu.edu.cn (G.Z.); 2 Intelligent Manufacturing Key Laboratory of Ministry of Education, Shantou University, Shantou 515063, China

**Keywords:** fatigue coefficient, stress concentration, notch coefficient, glass fiber, auto interior parts, virtual measurement, recognizable performance evaluation

## Abstract

This paper analyzes the structure of the key parts of the car belt guide, and the average stress of the vulnerable parts is simulated by analysis software. The theoretical stress of the section is calculated. The theoretical stress concentration factor ($K_{t}$) is given. The relation between the gap radius and the notch coefficient ($K_{f}$) was studied according to a previous $K_{f}$ calculation formula. The tensile tests of real products are used as reference data. The results showed that $K_{f}$ and $K_{t}$ are linear in most cases, but there are also cases of non-compliance. The relationship between the fatigue notch coefficient $K_{f}$ and the theoretical stress concentration coefficient $K_{t}$ was closely related to the service life and fatigue strength of the product. In addition, we found that the size and direction of warpage improved significantly with the increase of fillet size, which was not consistent with the effect of adding glass fiber material. The rounded corners of ordinary PP materials usually displayed forward warping, but the addition of glass fiber into PP materials made the degree of warping smaller, or even led to reverse warping. The size of rounded corners is an important optimization parameter. The relationship between $K_{f}$ and $K_{t}$ was studied from the perspectives of virtual measurement (VM) and recognizable performance evaluation (RPM). According to abnormal filling pressure, these relationships were compared with filling data to generate a fracture initiation control model. Based on a large amount of normal process data and quality inspection data, the historical data (causes) and quality inspection data (results) were combined.

## 1. Introduction

With the development of the economy, people’s living standards continue to improve, and the automotive industry is closely related to daily life. Various automotive interior parts in cars affect the experience of car owners and passengers. Automotive interior parts are mostly polymer parts, and mold flow analysis is critical to injection molding production. Mold flow analysis is the use of a computer numerical analogy method combined with finite body analysis method to solve the changes of various physical quantities of polymer melt in the mold cavity flow, pressure holding, and cooling, so as to obtain the temperature field and pressure field of polymer melt. The distribution of the velocity field can then predict various potential problems in the process of filling, holding pressure, and cooling. There are many problems in the products produced, and it is very necessary to optimize the analysis of existing products. The inevitable problem in the injection molding production process is that the injection-molded parts are defective. The problems caused by the filling, cooling, pressure holding, and warping steps will cause the product structure to appear with dangerous points, which will shorten the product life and optimize the product risk. The key to improving the quality of automotive interior trim parts is to select a better structure for injection production.

The existing injection molding production process is susceptible to the influence of materials, and the material has always been one of the keys to the performance of polymer parts, but the relationship between the defect analysis of some injection parts and the material is not necessary. We all know that in metal parts, polymer parts, or construction projects, gaps, even small gaps, will cause stress concentration. The direct response is to avoid this problem, but the different rounded corners have different effects. In addition, with the continuous advances in lightweight materials technology and the market demand, fiber-reinforced materials have emerged. Due to the microstructure (such as orientation, length, and concentration) of fiber-reinforced thermoplastics, their mechanical properties can be greatly improved compared to other injection-molded products. The fiber structure plays an important role in strengthening the mechanical properties of composites and has a certain influence on the stress concentration of components. This analysis is the key to this article. There are many gaps in injection molding production, which are located in uncritical positions and have little impact on the production parts. However, they are located in locations with high stress and have a great impact on the service life of injection-molded parts. Therefore, for the key positions of injection-molded parts, structural analysis, seeking the relationship between structure and quality, is very beneficial to the future production of the components.

In addition, the materials used in injection-molded parts are polymer materials, and the properties and structure of polymer materials are more complicated. Therefore, in this paper, research on polymer materials is not involved, so for the polymer materials, the research on the relationship between the theoretical stress concentration factor $K_{t}$ and the notch factor $K_{f}$ is very meaningful. In this paper, the selected automotive interior part, namely the automotive safety belt guide, is subjected to mechanical analysis and analysis of its key stress points, according to the definition of theoretical stress concentration coefficient $K_{t}$ and the results of Moldex3d software, to calculate the value of $K_{t}$. Then, this is used on a metal material to calculate the value of polymer materials of $K_{f}$ size. These two values are calculated respectively for the two groups of data. The data is then analyzed, and experiments are conducted to verify that the theoretical value is reasonable. In addition, we also studied the glass fiber and fillet size factors’ roles in producing the two defects studied: stress and warping deformations (defects).

## 2. Literature Review

A very important development direction in Industry 4.0 is computer-aided engineering (CAE) technology. For polymer production, the use of CAE technology to analyze and optimize polymer molding can avoid many production errors, thereby improving product quality. The advancement of finite element simulation technology provides a prerequisite for the optimization of injection molding production. The use of mold flow analysis software is a common application in injection molding production. Re-modeling converts product entities into software models that are then combined with CAE technology to obtain parameter data for filling, cooling, packing, warpage, and other processes in the analysis results. We analyzed the notch coefficient and stress concentration data, then performed data fitting to determining the relationship between the two.

In 2018, Maggipinto, M. et al. [1] mentioned that using the massive data collected by industry is definitely one of the main challenges of the so-called big data era. In this sense, machine learning is gaining more and more attention in the scientific community because it allows the extraction of valuable information through statistical prediction models trained on historical process data. In industrial manufacturing, one of the most widely used data-driven applications is virtual metering, in which expensive or unmeasurable variables are estimated through cheap and easily available measures already in the system.

The integration of the parameter-based machine response method and the material compressibility effect of the molten polymer in the simulation analysis is noted in this paper. By obtaining more accurate results from the injection pressure simulation, users are able to determine the dynamic behavior of the material more realistically as it enters the mold cavity. Through this integrated method, material waste in the mold test process can be decreased, and the challenges faced by front-line engineers in the actual process can be reduced.

The research on the relationship between the fatigue notch factor $K_{f}$ and the theoretical stress concentration factor $K_{t}$ focuses on metal. There are other relationships found in the literature, but in most cases, $K_{f}$ and $K_{t}$ have a linear relationship. There are only a few analysis reports for polymers that can control the fatigue notch coefficient of injection molding parameters. These relations have good applicability. This study uses these relations to study the relationship between $K_{f}$ and $K_{t}$ in injection-molded parts. In 2003, Makkonen, M. [2] mentioned that there are two types of gap-related size effects: statistical size effects and geometric size effects. The statistical size effect is calculated based on the maximum depth distribution of the initial crack in a specimen with varying stress regions.

In 2020, Liu, X. et al. [3] considered the geometric size effect and statistical size effect, and proposed the weakest link model for the calibration of probabilistic life prediction under low cycle fatigue (LCF). The weakest link model is first extended to the LCF state by using damage parameters and then calibrated based on the crack growth process. In 2020, Pellinen, H. [4] discussed digitalization in the structural design of robotic welding and how to consider digitalization in robotic welding. The structural shape of the joint and the end of the trapezoidal stiffener were studied by analytical calculation and the finite element method. The results showed that a 45° right-angle oblique angle is better than an arc-shaped oblique angle, and bending the tip of the stiffener can increase the fatigue life. In 2020, Jiao, Y.-n. et al. [5] studied the relationship between the number of cracks and stress, reported through statistical distribution. The cumulative probability of failure of cast aluminum alloys under a certain stress can be predicted by the combination of Paris’s law and pore size distribution. It was found that the depth of the pores dominates the stress field around the surface pores. When the pores and the surface were intercepted at the top, the maximum stress sharply increased. In 2020, Liao, D. et al. [6] mentioned that structural integrity assessment with discontinuities is essential to ensure the service life and reliability of engineering components. The experimental data of TA19 notched specimens of different scales were used for model verification and comparison. The correlation between the predicted fatigue life and the experiment produced by the proposed framework was acceptable.

In practical parts design, in addition to the best process parameters, good product structure and excellent materials are often complementary. They determine the molding quality of the parts. Adding glass fiber into plastic can not only reduce the weight of the product, but also improve the durability and impact resistance of the product, which is an effective way to improve the product performance without changing the product geometry. In 2015, do-Hyoung Kim et al. [7] designed and manufactured a glass/carbon composite hybrid bumper beam using glass/carbon felt thermoplastic (GCMT) composite material instead of traditional glass felt thermoplastic (GMT) to reduce the weight of the bumper beam. The optimized GCMT bumper beam was found to have a 33% reduction in weight and improved impact performance when compared to conventional GMT bumper beams. In 2017, Li Kun, Yan Shilin, Pan Wenfeng, and Zhao Gang [8] noted warpage as an important indicator to measure the quality of injection-molded parts of short-fiber-reinforced composites. In addition to normal process parameters, fiber parameters also have significant influence on warpage. In this study, the objective function was the smallest warpage problem. Design parameters included the fiber aspect ratio, fiber content, injection time, melt temperature, mold temperature, and pressure holding. In 2021, Collins Caroline et al. [9] reduced warpage by introducing rib features to increase component stiffness. They did not find any serious warping inside the parts, but the researchers suggested that the addition of ribs helps control the warping shape of the section.

Modern industries are actively taking advantage of the big data revolution to further expand smart manufacturing (SM) and virtual measurement (VM) strategies. This will improve integration and analysis capabilities, as well as providing solutions for predictive maintenance and optimized scheduling. Previously, model-based process control was applied to manufacturing technologies such as industrial machinery automation. By improving control and reducing quality variability, such as thickness and uniformity, model-based process control successfully proved its ability to improve the process. Currently, batch control is commonly used in most industries, and batch control is a necessary requirement for many advanced manufacturing processes. Various industries have successfully adopted model-based process control schemes, combining batch control, scheduling, and other functions.

In 2016, Tieng, H. et al. [10] stated that VM is a method to speculate on process tools based on the data sensed in process tools without physical measurement, knowledge of the method of manufacturing, or operation. In other words, the VM can convert sample inspections with metering delays into real-time and online total inspections. After completing the calculation of product quality and production efficiency in the virtual system, the data are reflected in the physical space as suggestions for further production. In the comparison between actual injection molding and simulation analysis, the most critical implementation step is to ensure that the input data of the simulation analysis are as consistent as possible with the conditions of the actual injection process. However, there are many factors that may lead to inconsistencies in subsequent comparisons, such as: mechanical properties in terms of faster or slower mechanical response times; characteristics in the material processing processes; the data measurement methods; and the geometric consistency of products. After ensuring the correctness of these input data, VM’s predicted results are usually highly consistent with actual results and can provide users with complete calculation data in the cavity to facilitate the optimization and adjustment of subsequent design changes. In 2018, Tieng, H. et al. [11] measured metering delay and severe part deformation. To overcome those two challenges related to the aerospace industry, that article proposed using an automatic virtual metering (AVM) system to successfully convert sample inspections with metering delays into real-time and online routine inspections. The article discussed the use of deformation fusion (DF) methods to deal with component deformation problems. In 2019, Chen, C.-C. et al. [12] used VM to monitor the electrical discharge machining (EDM) process: a probe with a very high sampling rate was required to obtain the voltage and current signals of the electrodes, so a large amount of sensor data were generated. When extracting features from raw sensor data, there may be significant data processing issues related to the sensor data. When there is an interaction between variables, the impact is greater than the impact of the original device/variable, and the original key variable search algorithm (KSA) solution may not be able to correctly find the root cause. In 2020, Sawatsubashi, T. et al. [13] from Mitsubishi Heavy Industries Co., Ltd. (MHI) studied the use of software sensors developed in cooperation with the University of Tokyo for the VM of power plant water quality feasibility. MHI developed a software sensor model, based on genetic-algorithm-based variable selection, nonlinear regression models, and adaptive models, that tracks changes in plant operation. The software sensor model verified that the chloride ion concentration, which is difficult to continuously measure, could be measured virtually and analyzed by using actual plant data. In 2020, Miao, Y. et al. [14] developed an automated technology that uses virtual reality (VR) equipment to measure eye deviation, where a VR display system in which a screen where the gaze target alternately changes between the on and off phases was used to simulate the normal strabismus diagnosis procedure. In 2020, Adnan, M. et al. [15] implemented systems with fast and intuitive capabilities that adapted to different metal properties, machine configurations, and process parameters. Therefore, it is challenging to determine the threshold of the main control loop of the additive manufacturing (AM) process. In 2020, Hsieh, Y.-M. et al. [16] ensured stable manufacturing and high output for factories (such as semiconductor or TFT-LCD injection molding plants) to carry out quality inspections on workpieces. Out of consideration for reducing the cost and cycle time, factories adopted sampling inspection, but due to the sampling strategy and measurement delay, real-time and online full inspection could not be realized. AVM is the best solution to solve the above-mentioned problems, because it can convert sampling inspection with metering delay into online real-time comprehensive inspections.

**Figure 1:**
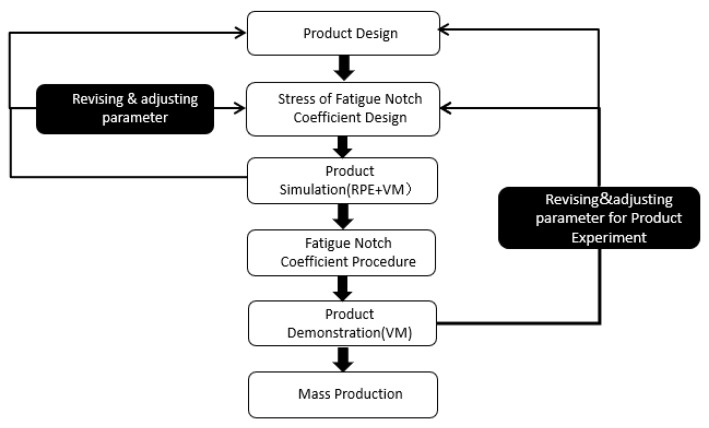
Flow chart using the recognizable performance of virtual measurement to ensure fatigue notch coefficient product quality.

Injection molding production is affected by a variety of factors, so the gaps produced by interior trim parts of the same structure are different. There are many examples of research on the notch coefficient and stress concentration of metal parts. For different types of notch conditions and materials, significant research has been completed, including research on the point stress model, the fracture mechanics model, and the field strength method model. The models have many generalized fitting formulas, but their applicability is different; in many cases, they need to be analyzed again. Only individual research formulas have had good applicability, but for automotive interior polymer parts, there is not much research literature on trims. For the fatigue notch coefficient and theoretical stress concentration, most of the research is to determine the value of the notch coefficient, perform stress tests, and obtain the relationship between the two by data fitting to make targeted improvements to the production situation of the notch and then screen the relationship between notch coefficient and stress concentration to obtain automotive interior parts with excellent fatigue strength. In this process, it is necessary to analyze the relationship between different notches and stress concentration. Therefore, we tried to obtain unprocessed design parameter values through VMM and identifiable performance evaluation (RPE) and then use systematic and effective experimental methods to determine the best combination of parameters. The execution process is shown in Figure 1.

The estimation of “hybrid” key weights (for defect factors) is something experts are reluctant to try because of “key defect weights that cannot be identified in the physical experiment formula”. Taking a non-special neural genetic network analysis as an example, experienced researchers have always known that it is important to be able to recognize (or identify) key weight values.

In 2017, Han-Jui Chang [17] proposed an applied anisotropic diffusion model to consider the interweaving of different factors and to evaluate its performance. When the parameter appears, the experimenter can discuss the phenomenon that appears. The iterative calculation of the post-processing RPE method can determine the interoperability of different physical properties and propose optimizations.

In 2019, Chang Han-jui [18] reviewed various methods for evaluating the performance of five-axis machine tools through identification and multi-type comparison, and RPE was found to be the most practical. RPE is currently one of the research methods that can obtain accurate reference data through quantitative and identification methods, and it is also one of the evaluation methods for multi-type five-axis machine tool methods. Based on the interface of RPE and IT level distribution in general mechanical design changes, this paper attempted to introduce fuzzy theory to obtain excellent research results. This study calculated the attribution level of the tested items. In view of the conflicts in the re-evaluation of the performance of various types of five-axis machine tools, this research method directly distinguishes and defuzzifies the attribution reduction interval and can directly judge and evaluate the prediction results.

However, according to the RPE method for identification, there are basically three defect factors: barrel temperature, back pressure, and V/P points. Preliminary experimental identification is prone to doubt. As there are many defects in injection molding, developers who use nominal is best calculations can easily be prone to cognitive uncertainty. Therefore, there are two or more connected features: either nominal + small is the best feature, or nominal + large is the best feature. In addition, there are blind spots in the physical experiment formula. In 2021, Han-Jui Chang [19] used the identifiable method of photoelastic stress for measurement and compensation combined with fuzzy theory to reorganize a set of processes that can be used to evaluate product residual stress. The corresponding theoretical formula can be used to effectively quantify and compensate for the measurement of the residual stress of the product. In 2021, Han-Jui Chang [20] pointed out that in the evaluation of identifiability, as it helps to make the product’s characteristics more prominent. It is not easy to evaluate the reference value due to the influence of different factors, but a single variable can become the dependent variable, the main influencing factor. With this information, users can discuss when this phenomenon occurs. In addition, a research and implementation method of defect knowledge based on VM was also proposed to realize an identifiable evaluation-independent injection molding quality control system. In the era of information and technology revolution, machines can generate and record data during the production cycle. Through big data management, data can now be processed to obtain troubleshooting methods.

In 2020, Han-Jui Chang [21] explained the concept of interaction effect in physics, when there are two or more independent variables in experimental research, and how one of the effects is produced. The independent variable exhibits alternation with another independent variable. The true influence of some factors are changed by another factor. The interaction between the warping directions of glass fibers was quantified. This method provided reference data and provided a simple method to determine whether it affected the mold (depending on whether glass fiber was added) and the holding time conditions. The experimental results showed that the warpage of injection-molded parts are influenced by the interaction of glass fiber content and holding time. Adding a certain amount of glass fiber into PP material and setting the appropriate compression time can greatly reduce the product warpage.

According to the above viewpoints, adding a certain amount of glass fiber into PP polymer plastics can improve the effect of warpage defects. For example, ordinary PP material injection products will have the defect of positive warpage, and the use of glass-fiber-reinforced PP material injection products can effectively reduce the size of warpage and even achieve the effect of reverse warpage, but high-quality materials are often accompanied by manufacturing difficulties and high costs. Therefore, from the point of view of geometric structure, this study proposes the idea of changing the fillet radius to improve the warping size and the reversal of the fillet. It is verified by modeling, software simulation analysis, and experimentation. In addition, based on a large amount of normal process data and quality inspection data, a control model is built by using the VMM and RPE to realize online real-time monitoring and quality prediction of products. 

## 3. Methodology

**Figure 2:**
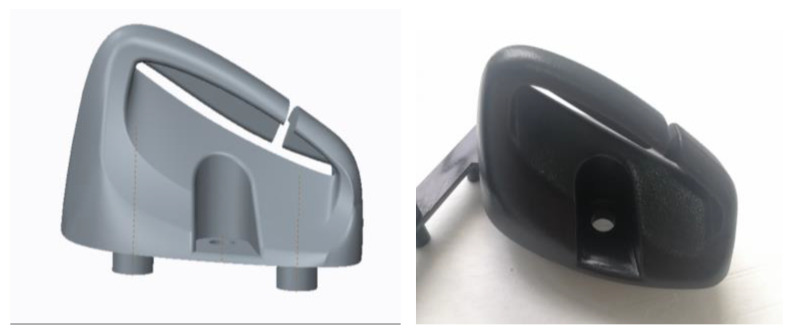
Auto interior part—seat belt guider sample images.

In this article, the vehicle interior parts shown in Figure 2 are used for analysis. The object is named the seat belt guide, and its function is to change the direction of the seat belt and connect the seat belt. Firstly, 3D modeling software (Creo2.0) is used for product modeling. Simulation software is used to simulate and analyze the product model to obtain the simulation mechanical data. Then, SolidWorks software is used to obtain the volume, quality, and cross-sectional area of the product model. According to the data, the value of $K_{t}$ is obtained according to the definition formula of the theoretical stress concentration coefficient $K_{t}$, the value of $K_{f}$ is obtained according to the calculation formula of the notch coefficient $K_{f}$ proposed by Peterson RE, and then the relationship between the two is analyzed through data fitting. In addition, according to the definition, the main parameter affecting the $K_{t}$ value is the crack depth at the key point of the model at the initial stage of stress. Besides the gap radius and material constant, the key data affecting the $K_{f}$ value are also directly related to the actual size of $K_{t}$. $K_{t}$ is actually related to the initial crack depth, so the notch coefficient $K_{f}$ value is also related to the initial crack depth. The main purpose of the research in this article is to study the influence of the notch radius. For the material constant, a planned value will be used for analysis as a constant. For the notch radius, two groups of different notch radii are used to analyze the $K_{f}$. Then, the respective fittings are performed, the two situations are compared, and the relationship between the notch radius and the notch coefficient $K_{f}$ is obtained.

The theoretical stress concentration factor $K_{t}$ is defined as the ratio of the actual stress of the section at the notch or other stress concentration points to the theoretical stress of the section. There are many definitions of the theoretical stress of the cross-section. Here, the average normal stress is obtained by dividing the axial load on the specimen by the net cross-sectional area of the notch. The actual stress of the section refers to the ratio of the effective stress corresponding to the dangerous surface of the test piece to the area of the section in the simulation of the actual injection molding production process.

For the theoretical stress concentration factor, it is necessary to take the theoretical value of the effective stress. The calculation of the theoretical effective stress is related to the crack depth in the notch. To obtain the crack depth, it is necessary to obtain the sample size and the number of initial cracks in the sample. The exact number of cracks is not known; however, the possible error of this value has no significant effect on the calculation result, so we refer to a value of 100 cracks per mm^2^ [2]. With the help of linear elastic fracture mechanics (LEFM), the required connection between the fatigue limit and the initial crack depth can be established (1) as follows:

(1) $$K_{I}=\beta б\sqrt{\pi a_{0}}$$

$K_{I}$: the fatigue limit
*β*: geometric factor
б: pressure
$a_{0}$: initial crack depth

In the definition of the geometric size effect, in a specimen of the same shape, the critical crack depth is smaller in the larger specimen, which also means a lower fatigue limit. However, under general conditions, it is very difficult to test the fatigue limit for a period of time, and this takes a large amount of time. Therefore, it is not feasible to directly measure the fatigue limit, and fatigue limit measurement cannot be completed at one time. This is very important for the data. It is fatal in terms of rigor. Therefore, we obtain the value of the average fatigue limit.

The geometric factor *β* depends on the sample shape, crack shape, and stress distribution. It is proposed that the geometric factor of constant stress is *β* = 0.735, and the corresponding aspect ratio a/c = 0.8 (a is the crack depth, and c is the crack length). The force of the car seat belt guider is also included in this study. The force is in a constant state, so the geometric factor *β* = 0.735 is cited [2].

Substituting the stress intensity range threshold Δ*K_I,th_* and the average fatigue limit Δб*_R_*_,a_ instead of KI, we obtain the following:

(2) $$\sqrt{a_{0}}=\frac{1}{\sqrt{\pi }}\left(\frac{\Delta K_{I,th}}{\beta {\Delta б}_{R,a}}\right)$$

$\Delta K_{I,th}$: stress intensity range threshold
${\Delta б}_{R,a}$: average fatigue limit

When the prediction of crack depth is available, the estimated value of the average fatigue limit can be obtained by the following equation:

(3) $$\frac{\Delta {б}_{R,a}}{2}=\frac{1}{\sqrt{\pi }}\left(\frac{\Delta K_{I,th}}{\beta \sqrt{a_{0}}}\right)$$

**Figure 3:**
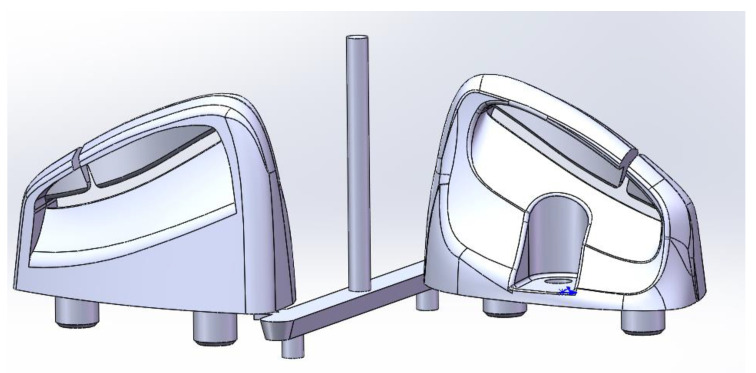
Auto interior part—seat belt guider sample diagram by Creo 2.0.

We divide the left-hand side by 2, because when calculating the stress intensity range, only the stretched part of the stress cycle is considered effective. For the material in question, the exact value of the stress intensity range threshold Δ*K_I,th_* is unknown. The length of the fillet of 0.46 mm radius is 120.27 mm (the bottom position is measured as 15 mm), and the length of the fillet of 0.52 mm radius is 120.89 mm (as Figure 3).

There are two models established by Creo 2.0. One model has a corner radius of 0.46 mm at the dangerous point, and the other model has a corner radius of 0.52 mm. Both models are assumed to be subjected to a constant pressure in the calculation, so referring to a/c = 0.8, each can be used. When the fillet radius is 0.46 mm (i.e., c = 0.46 mm), the size of crack depth a is 0.368 mm; when the fillet radius is 0.51 mm (i.e., c = 0.51 mm), the size of crack depth a is 0.408 mm.

The crack depth values of both are average values, which are in a stable state, and the initial crack depth value should be smaller than the average value. Assuming that the model with a fillet radius of 0.46 mm is stressed, the initial crack depth is 0.33 mm or 0.35 mm; after the model with a fillet radius of 0.52 mm is stressed, the initial crack depth is 0.38 mm or 0.40 mm. Then, the corresponding force can be brought into Equation (3) and Equation (1).

### 3.1. Calculation of the Theoretical Stress Concentration Factor

**Figure 4:**
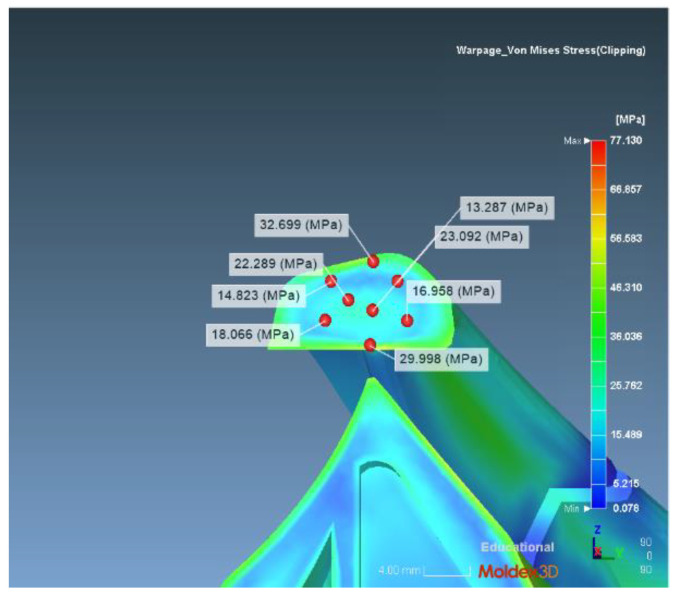
Distribution of the 0.46 radius theoretical stress concentration factor.

(1) Calculation of the theoretical stress concentration factor with a notch radius of 0.46. The actual stress of the section is determined by mold flow analysis using Moldex 3D software, and then the actual stress of the corresponding section is determined. On the corresponding section, we randomly select eight points, as shown in Figure 4, and the resulting stress levels are: 14.823 MPa, 22.289 MPa, 18.066 MPa, 16.958 MPa, 32.699 MPa, 23.092 MPa, 29.998 MPa, and d13.287 MPa. We take the average value as the actual stress of the section: 21.402 MPa.

**Figure 5:**
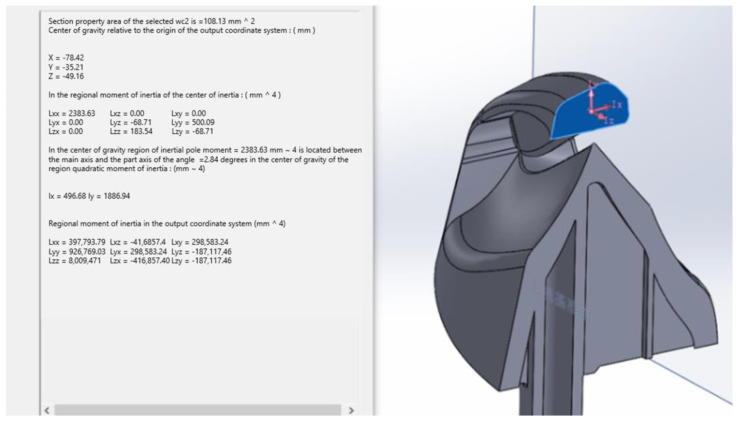
Resolution of the 0.46 radius theoretical stress concentration factor.

The theoretical cross-section stress refers to the average normal stress obtained by dividing the axial load on the specimen by the net cross-sectional area of the notch. The cross-section is irregular, and its area cannot be obtained by direct calculation. Using SolidWorks’ function of evaluating section properties, the area of the cross-section is measured. The test result is shown in Figure 5. The cross-sectional area of the theoretical stress of the cross-section is 108.13 mm^2^, the axial load is preset to 234.973 N, and the theoretical stress is obtained as 2.173 N/mm^2^, which is converted into 2.173 MPa. Therefore, the value of the theoretical stress concentration factor $K_{t}$ is 9.989. The size of the axial load is preset to 221.546 N, and the theoretical stress is obtained as 2.049 N/mm^2^, which is converted to 2.049 MPa. Therefore, the value of the theoretical stress concentration factor $K_{t}$ is 10.445.

**Figure 6:**
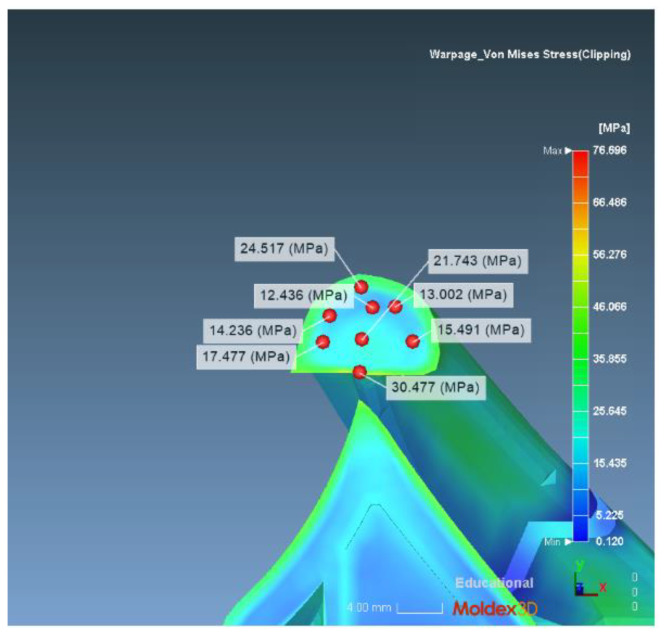
Distribution of the 0.51 radius theoretical stress concentration factor.

(2) Calculation of the theoretical stress concentration factor with a notch radius of 0.51 mm. Similarly, we select eight points on the model section with a notch radius of 0.51 mm, as shown in Figure 6. The resulting stress levels are: 24.517 MPa, 14.236 MPa, 15.491 MPa, 21.743 MPa, 12.436 MPa, 17.477 MPa, 13.002 MPa, and 30.477 MPa. We take its average value: 18.297 MPa.

**Figure 7:**
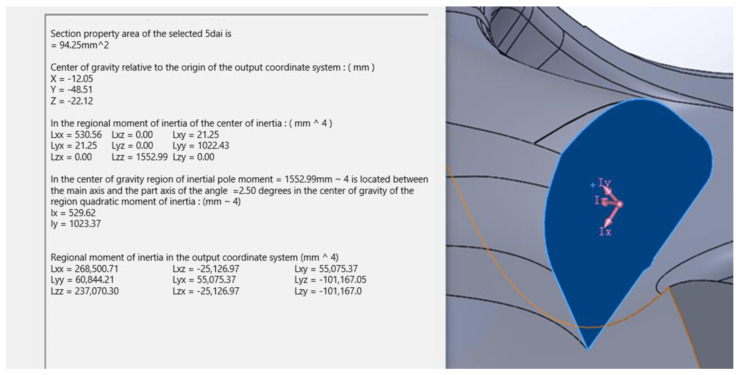
Resolution of the 0.51 radius theoretical stress concentration factor.

The cross-sectional area is also obtained through the evaluation section function of SolidWorks. The cross-sectional area is 94.25 mm^2^. As shown in Figure 7, the axial load is preset to 210.112 N, and the theoretical stress is 2.229 N. The mm^2^ is converted into 2.229 MPa. Therefore, the value of the theoretical stress concentration factor $K_{t}$ is 8.209. The size of the axial load is preset to 204.653 N, and the theoretical stress is obtained as 2.171 N/mm^2^, which is converted to 2.171 MPa. Therefore, the value of the theoretical stress concentration factor $K_{t}$ is 8.428, as shown in Figure 6 and Table 1.

The two models used for calculation have the same material and functional structure except for the slight shape error in the modeling. The difference is mainly in the key points with different fillet radii. Obviously, it can be considered that the two models belong to the same parent body. In addition, in the same model, the size of the initial crack depth affects the size of the average fatigue limit. The smaller the initial crack depth, the greater the average fatigue strength, and the greater the pressure it receives. The theoretical stress concentration factor $K_{t}$ has a negative correlation with the initial crack depth. In addition, the position of the point on the cross-section of the key point also affects the magnitude of the theoretical stress concentration factor $K_{t}$.

**Figure 8:**
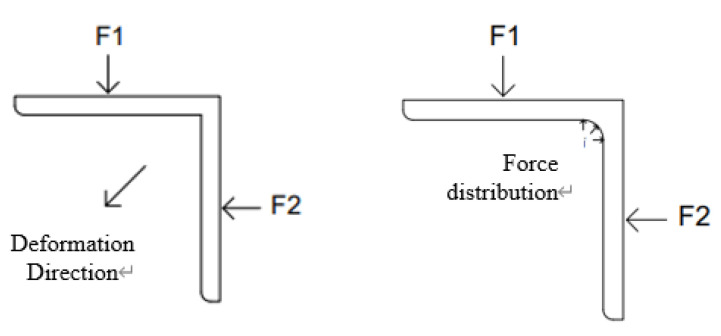
Stress concentration avoidance by rounded structure.

In a structure with a large force, the junction is the dangerous point of the structure, and the joint action point of the two moments is located at the junction. If a fracture occurs, it is often the dangerous point that breaks first. Because it is at the intersection of two forces, this situation is called stress concentration. A rounded structure can avoid stress concentration. When the force is concentrated in a rounded structure, the force is no longer concentrated on the junction but distributed on the arc surface of the rounded corner. The force at one place will not be too large, thus avoiding stress concentration. As shown in Figure 8 below, when the component receives downward force and leftward force, the component will deform to the lower left corner. If it is a right-angle structure, the force will be concentrated at one point. The composition is also located at a point, so it is easy to destroy the location of this point. If it is a rounded structure, then the force will be fan-shaped, distributed on the rounded corners. The force on the fillet will not be concentrated at one point, and it is not easy to damage the junction. In turn, the fatigue strength is prevented from being reduced.

The rounded corner structure is often used to avoid stress concentration. There are also designs that are designed to be more beautiful, where outer rounded corners are used to avoid excessively sharp parts. Different fillet radii can achieve different levels of mechanical dispersion. If the junction is regarded as a gap, then the design of the fillet should reduce the size of the notch coefficient, because the size of the notch coefficient is related to the radius of the fillet.

### 3.2. Calculation of Gap Coefficient

The finite life fatigue strength or infinite life fatigue limit of notched specimens is affected by the concentration of notched stress, which is obviously lower than the fatigue strength or fatigue limit of smooth specimens. This phenomenon is characterized by the fatigue notched coefficient $K_{f}$. Since $K_{f}$ was proposed, there have been a large number of summarized empirical formulas or semi-empirical formulas, which correspond to the formulas studied in different situations. The applicability of each formula is different, which indicates that the foundation of these formulas is not solid. The size of the notch coefficient is affected by the material, and material is a very complex problem. Most existing formulas are for certain materials, or the material constant is calculated as a fixed constant value.

For the relationship between $K_{t}$ and $K_{f}$, the value of $K_{t}$ can be obtained by calculating the ratio of the theoretical effective stress to the actual effective stress of the dangerous section according to the definition, but a more rigorous method for the value of $K_{f}$ should be found. A large number of experiments are performed to measure the $K_{f}$ values of different gaps, and the relationship between $K_{t}$ and $K_{f}$ is then obtained through linear fitting. Linear fitting analysis of the relationship between $K_{t}$ and $K_{f}$ is generally achievable, and most materials satisfy $K_{t}$ and $K_{f}$. $K_{f}$ has a linear relationship, but the large number of experiments to determine $K_{f}$ require certain experimental conditions, and the determination of the fatigue limit requires an extremely long time to complete. Therefore, this article chooses to use the existing well-applied formula to calculate the value of $K_{f}$.

There is a large amount of research on the fatigue notch coefficient in the aerospace sector, and planes need a high fatigue strength to complete flight missions in high altitude. There are many studies on the metal notch coefficient of the aircraft. The research in this area is based on a large amount of data for fitting analysis. There is also a lot of research on composite materials. The calculation formula of $K_{f}$ can be divided into the point stress model, fracture mechanics model, and field strength method model. These three models cover many $K_{f}$ expressions.

The point stress model, which is more practical in engineering, is represented by the local strain method. Based on the idea of the structural unit, it is considered that a structural unit with a length of A can be measured inward from the root of the cut on the plane of the smallest section. Before cracking occurs, the average loop stress on this length must exceed that of the smooth specimen of the material. Regarding the fatigue limit, based on the idea of the structural unit, the structural unit of length A can be measured inward from the root of the incision on the smallest section plane [2].

(4) $$K_{f}=1+\frac{Kt-1}{1+\left[\pi /\left(\pi -\omega \right)\right]\sqrt{A/\rho }}$$

ρ is the radius of the notch root, and *π*/(*π* − *ω*) is the correction material constant that takes into account the influence of the notch opening angle. *A* is a function of tensile strength, and the balance is between 0.51 mm and 0.025 mm. Assuming that the stress decreases linearly from the root of the notch inward, it is obtained by considering the support effect of the material with relatively low subsurface stress on the high-stress material.

(5) $$K_{f}\;=1+\frac{K_{t}-1}{1+\alpha /\rho }$$

α: material constant
ρ: notch radius

Regarding the material constant, the core of the research in this paper is not the material, so the material constant is set to a constant value, and the constant value is 1. The formula becomes as follows:

(6) $$K_{f}\;=1+\frac{K_{t}-1}{1+1/\rho }$$

The theoretical stress concentration factor $K_{t}$ of the above two models with a notch radius of 0.46 mm and a notch radius of 0.51 mm has been calculated. The $K_{t}$ value of the model with a notch radius of 0.46 mm (a_0_ = 0.33) is 14.024, and the notch radius is 0.46. The $K_{t}$ value of the 0.51 mm (a_0_ = 0.35) model is 14.873, the $K_{t}$ value of the model with a notch radius of 0.51 mm (a_0_ = 0.37) is 8.209, and the $K_{t}$ value of the model with a notch radius of 0.51 mm (a_0_ = 0.39) is 8.428. We substitute the theoretical stress concentration factor and the notch radius into the formula of $K_{f}$ and obtain the following:

ρ = 0.46 mm (a_0_ = 0.33):

$$K_{f}\;=1+\frac{K_{t}-1}{1+1/\rho }\;=1+\frac{9.989-1}{1+1/0.46}=2.832$$

ρ = 0.46 mm (a_0_ = 0.35):

$$K_{f}=1+\frac{K_{t}-1}{1+1/\rho }\;=1+\frac{10.445-1}{1+1/0.46}=2.976$$

ρ = 0.51 mm (a_0_ = 0.37):

$$K_{f}\;=1+\frac{K_{t}-1}{1+1/\rho }\;=1+\frac{8.029-1}{1+1/0.51}=2.374$$

ρ = 0.51 mm (a_0_ = 0.39):

$$K_{f}\;=1+\frac{K_{t}-1}{1+1/\rho }\;=1+\frac{8.428-1}{1+1/0.51}=2.509$$

The data is organized as shown in Table 2.

## 4. Materials and Methods

**Figure 9:**
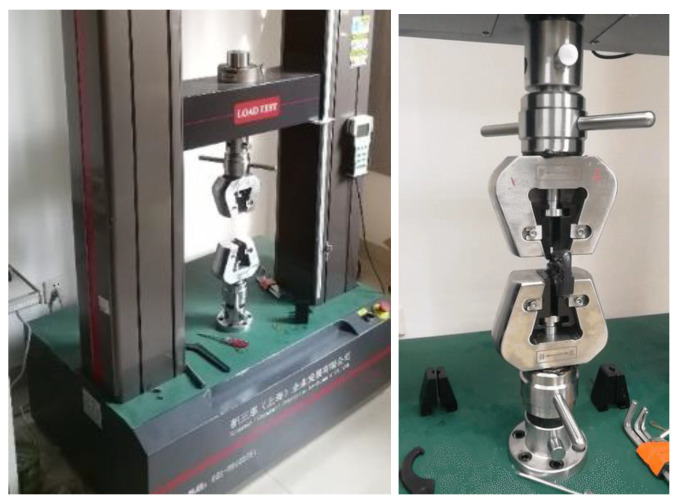
Tensile strength of experimental specimen.

After completing the theoretical analysis, a batch test tensile strength experiment was carried out in the laboratory, using the material tensile testing machine shown in Figure 9. After conducting multiple tests, the results of the experiment are as shown in Table 3, that is, the tensile strength is 238.04 N.

Using theoretical calculations, the cross-sectional areas corresponding to the 0.46 mm and 0.51 mm models are 108.13 mm^2^ and 94.25 mm^2^, respectively, as shown in Table 4:

There is little difference between the two sets of tensile strength data, so it is feasible to use them as the calculation basis. The theoretical values of $K_{t}$ and $K_{f}$ are not much different from the $K_{t}$ and $K_{f}$ values of the tensile strength obtained from the actual experiment. It can be considered that the PP material also conforms to the relationship between $K_{t}$ and $K_{f}$. Here, the effective stress of the thermal residual stress on the software is obtained. On the one hand, the theoretical effective stress can be obtained by setting the force on the section and the ratio of the force to the section area, which is the theoretical effective stress. The direct setting of force needs to be based on reliable values. Therefore, referring to the method of calculating the crack depth, there is a reasonable preset value for the initial pressure, and the theoretical stress concentration factor $K_{t}$ is calculated.

The cause of the notch factor $K_{f}$ is that in some structural designs, grooves, shoulders, and other structures are prone to appear. These structures are prone to stress concentration. However, the research object in this paper uses rounded corners to avoid stress concentration. The above is to satisfy the notch coefficient, and the rounded corners are also a kind of notch. The experimental results are in line with expectations.

## 5. Demonstration of Virtual Recognizable Performance Evaluation

According to the deformation displacement data, we compare the abnormal fillet radius. The process data of different time periods (theoretical design and variant displacement) were compared to determine which variant displacement control parameters have changed during normal and abnormal time periods. In view of the problem that the internal fasteners do not have a batch number, and therefore cannot be tracked according to the quality of the batch, we have analyzed and determined several control variables with the highest similarity to the variation displacement change law and determined that the process parameter changes according to the variation. The time varies between detections. Based on the above analysis, the specific and maximum suspected circle radius and pressure density value control parameters that caused the deformation displacement were automatically located and determined by the process engineer. The above analysis helped the process engineer to quickly locate the cause of the problem. Based on historical circle radius data and pressure density quality inspection data, a control model between the two is established. Based on a large amount of normal process data and quality inspection data, the historical data (causes) and quality inspection data (results) were combined in the equation function.

Two things can be done on the VM function:

(1) Quantitative parameter adjustment: When warpage occurs, according to the target value of the specific variant, the adjustment range corresponding to the corresponding pressure density value parameter is deduced for precise parameter adjustment;
(2) Mapping measurement: According to the current data of the pressure density value control parameter, based on the mapping function, real-time prediction of the variant value over the next few hours can be accomplished. Once the variant exceeds the preset threshold, it will immediately warn or even stop production.

**Figure 10:**
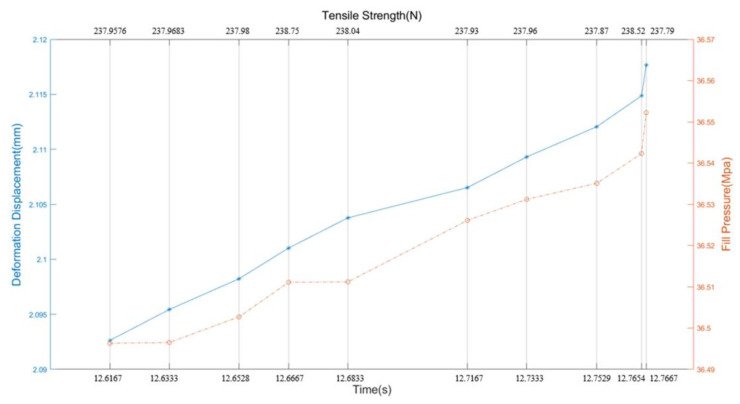
Batch control “deformation displacement” shows that the deformation displacement is close to the variation of the filling pressure value, both of which are helpful to improve the “tensile stress data”.

Through the collection of a large amount of real-time equipment data and quality inspection data, the mapping relationship indicating causality is constructed through data analysis and modeling. Current parameters that can be directly measured are calculated in the present to find abnormalities and defects that can only be found in the inspection process in the future; through the current measurement values, the indicators that cannot be directly measured, such as the filling pressure value (VP switching point), the center temperature of the filling position, and the abnormal displacement of the deformation, can be indirectly inferred. As shown in Figure 10, it can be deduced that according to the deformation displacement data, the abnormal filling pressure is in a positive relationship. This can also be used to effectively schedule and dispatch the plan and adopt configurable control rules to simulate the process in the injection molding factory. The product process is used to determine how to make decisions in the scheduling of the entire injection molding plant and the scheduling of specific machines. For example, by understanding the length of queue waiting time, production cycle, delivery time, and combining product priorities (such as emergency batches), real-time scheduling can be enabled, and control rules can be used to optimize the working process to achieve customer satisfaction. Yield targets are shown in Table 5. These rules can be easily updated and reset to respond to changing or unpredictable situations (such as changing needs or unexpected downtime). These functions and other innovations have enabled the industry to continue to take the lead in this technology field, and other vertical industries with strict process control requirements are also considering the use of real-time scheduling and scheduling solutions.

## 6. Discussion and Result

Here, we look at the difference between polymer PP materials (no glass fiber) and polymer PP materials (with glass fiber).

This section will compare the two parameters of $K_{t}$ and $K_{f}$ between polymer PP material without glass fiber and polymer PP material with glass fiber. In the above calculation, the parameters of common polymer PP material are mainly concerned with the initial crack depth and density. However, under the condition that the fillet radius remains unchanged (0.46 mm and 0.51 mm) and the dangerous section load remains unchanged, only the polymer PP material containing glass fiber is expanded. Firstly, the $K_{t}$ of polymer PP material containing glass fiber is calculated, and the results are shown in Table 6.

We substitute the theoretical stress concentration factor and the notch radius formula to obtain following:

ρ = 0.46 mm (a_0_ = 0.33):

$$K_{f}=1+\frac{K_{t}-1}{1+1/\rho }\;=1+\frac{6.670-1}{1+1/0.46}=1.786$$

ρ = 0.46 mm (a_0_ = 0.35):

$$K_{f}=1+\frac{K_{t}-1}{1+1/\rho }=1+\frac{7.074-1}{1+1/0.46}=1.914$$

ρ = 0.51 mm (a_0_ = 0.37):

$$K_{f}=1+\frac{K_{t}-1}{1+1/\rho }=1+\frac{5.917-1}{1+1/0.51})=1.661$$

ρ = 0.51 mm (a_0_ = 0.39):

$$K_{f}\;=1+\frac{K_{t}-1}{1+1/\rho }=1+\frac{6.075-1}{1+1/0.51}=1.714$$

Data are organized as shown in Table 7.

**Figure 11:**
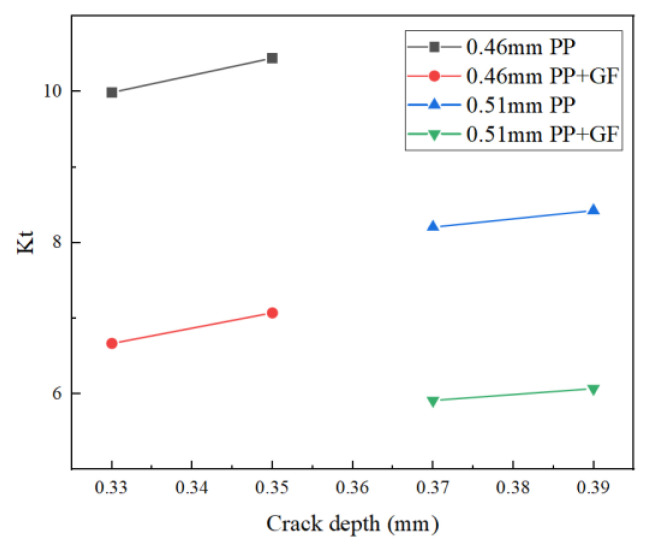
Theoretical stress concentration coefficient of PP materials (with glass fiber) and PP materials (no glass fiber).

**Figure 12:**
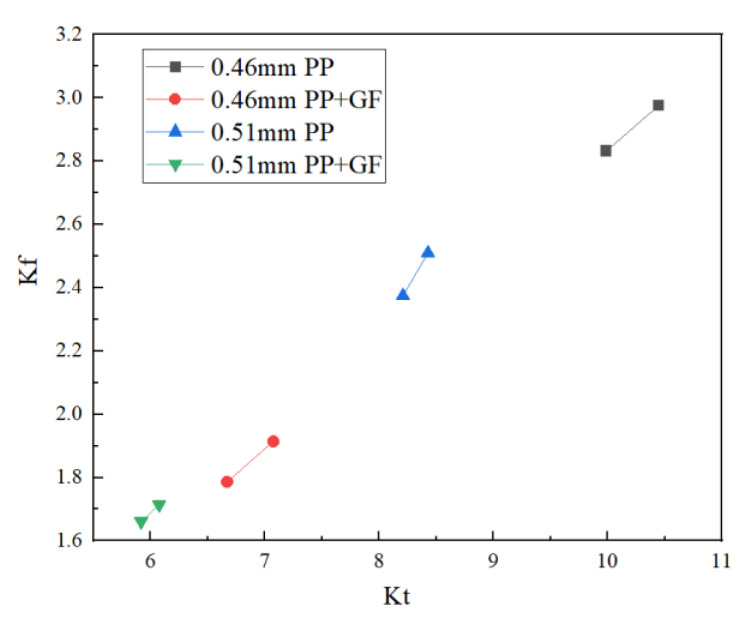
$K_{t}$ and $K_{f}$ relationship trend.

A comparison table of theoretical stress concentration factor $K_{t}$ and notch factor $K_{f}$ between polymer PP materials with no glass fiber and polymer PP materials with glass fiber is shown in Table 8, and a trend chart is included in Figure 11 and Figure 12.

According to the above results, we can determine that in the case of the same size of material and rounded corner, $K_{t}$ increases with the increase of crack depth. To be precise, there is a positive correlation. When the rounded corners are of the same size, the $K_{t}$ value decreases with the addition of glass fiber. In the case of the same material, the value of $K_{t}$ will also decrease with the increase of rounded corner size and crack depth. For PP materials doped with glass fiber, the size of the gap coefficient $K_{f}$ increases with the increase of theoretical stress concentration factor $K_{t}$. Although there are only two sets of data, this can be roughly described as a linear correlation. In the trend diagram, initial crack depth a_0_ and the relationship between the stress concentration factor $K_{t}$ can also be regarded as a linear correlation. The relationship between $K_{t}$ and $K_{f}$ is also more linearly correlated, and the greater the change of $K_{t}$ value, the greater the change threshold of $K_{f}$.

**Figure 13:**
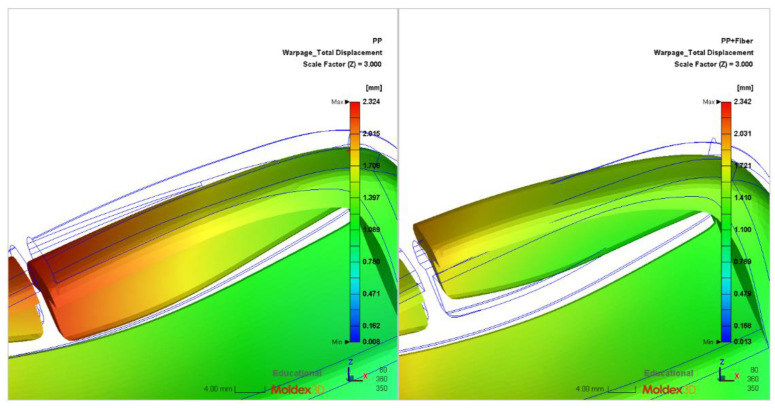
Simulation analysis results of different glass fiber warping orientations at round corners.

By adding fiber into the pure polymer material, the warpage of the product at the rounded corner can be improved. As shown in Figure 13, the figure on the left is the simulation analysis diagram of pure PP material’s warping deformation at the fillet after amplification of three times. The results show that the warping deformation at the fillet of pure PP material is notable, and the warping direction is in the direction of the fillet. Comparing this with the PP material containing glass fiber, in the figure on the right, it can be seen that the addition of glass fiber can significantly improve the size of the warpage at the rounded corner, most of the product shape can be well maintained, and a small part of the warpage direction is deformed toward the rounded corner. In other words, the addition of glass fiber can effectively improve the structural strength of parts and can greatly improve the size and direction of warping deformation. In addition, the fiber orientation has an important influence on the warpage of parts. When uneven fiber orientation causes large warpage deformation of products, the fiber orientation can be adjusted by optimizing the gate position and product structure to reduce the warpage deformation. In addition, with the increase of fiber content, the difficulty of plastic filling flow will obviously increase, so in order to ensure smooth plastic filling the mold cavity, the injection pressure and injection speed should be increased.

Increasing the holding time can reduce the warping deformation and residual stress of injection-molded products, so as to improve their structural strength. Constant pressure holding will cause the cavity pressure to become very uneven, which can easily produce large residual stress. The pressure holding process setting will make the mold cavity pressure more average, that is, it can reduce the uneven shrinkage of plastic parts and can reduce the pressure in the mold cavity, improve the molding quality of plastic parts, and achieve the purpose of reducing the warpage deformation of plastic parts.

As can be seen from the comparison table, regardless of whether the PP material contains glass fiber or not, the size of the notch coefficient $K_{f}$ will increase with the increase of the theoretical stress concentration coefficient $K_{t}$. Although there are only two sets of data, it can be roughly considered that the two are linearly correlated. In the trend diagram, the relationship between initial crack depth a_0_ and the theoretical stress concentration coefficient $K_{t}$ can be seen as a linear correlation, and the relationship between $K_{t}$ and $K_{f}$ can be seen as more linear. The greater the change of $K_{t}$, the greater the change threshold of $K_{f}$.

This section looks at the fillet sizes of 0.46 mm and 0.51 mm and whether PP material contains glass fiber as the influencing factors to conduct the following four groups of control tests: (1) 0.46 mm rounded corner structure and material PP; (2) 0.46 mm rounded corner structure and material containing glass fiber PP; (3) 0.51 mm rounded corner structure and material PP; (4) 0.51 mm rounded corner structure and material containing glass fiber PP. The simulation of product molding is carried out by using analysis software, and the stress value and warpage deformation at the fillet are analyzed as quality indicators. The test results are shown in Table 9.

PP material added to the glass fiber can significantly reduce the size of the warpage parts and the stress value of the gap. Compared with the rounded structure with a radius of 0.46 mm, the stress concentration of the rounded structure with a radius of 0.51 mm is smaller. By increasing the rounded radius, the stress at the crack is reduced by about 21%. Warping at 0.46 mm rounded corners has been reduced from 1.3950 mm to 1.368 mm, and the warping at 0.51 mm rounded corners has been reduced from 1.4318 mm to 1.288 mm. It can be seen that increasing the fillet size and adding glass fiber can reduce the stress value and warping during fillet removal.

Overall, optimizing the structure of the product (increasing the radius size) or using more advanced and superior materials (in PP polymer materials, such as glass fiber) can improve the gaps, to a certain degree, with regard to the stress concentration phenomenon. However, there are often more manufacturing problems with high-quality material, such as difficulty and expense, and optimizing the product structure design is often cheaper. Therefore, the larger fillet radius can reduce the warping and notch stress and solve some necessary engineering problems.

## 7. Conclusions

The car seat belt guider selected in this article has rounded corners due to its design, which is very similar to what is used in the research on the metal notch coefficient. Notches have the phenomenon of stress concentration, so the existence of some small notches will greatly reduce the service life and fatigue limit of the component. In this paper, the theoretical stress concentration coefficient $K_{t}$ and notch coefficient $K_{f}$ of the car seat belt guide with a rounded corner structure were studied. In addition, the stress concentration distribution and warpage defect were also studied with or without mixed glass fiber and different rounded corner size. For notch stress analysis, injection molding can use molding parameters to adjust the internal pressure and density, which is the biggest advantage.

The results showed that for 0.46 mm and 0.51 mm rounded corners, the addition of glass fiber reduced the stress at the rounded corners by 32% and 18%, respectively, improving the stress concentration. The addition of glass fiber can improve the toughness and stiffness of PP, thus reducing the stress value at the rounded corners of PP. For round corner warping, the glass fiber affects the forming of parts and improves the final warping deformation of products. The addition of glass fiber reduced the warping at rounded corners by 1.94% and 10%. Compared with forward warping without glass fiber, glass fiber can reduce the degree of warping and even achieve the effect of reverse warping.

Whether the material uses contains glass fiber or not, the larger the size of the workpiece fillet, the smaller the stress value at the fillet. The fillet size also has an influence on warpage value and stress value. Parts with less fiber and 0.46 mm rounded corners were the worst, while those with fiberglass and 0.51 mm rounded corners were the best.

A lot of research has been done in the automotive field, and the relationship between the theoretical stress concentration factor $K_{t}$ and the notch factor $K_{f}$ has been summarized for different materials, different shapes of gaps, and different models, and the calculated formula is of great benefit for the subsequent research. Especially in the calculation of the gap coefficient $K_{f}$, many inductive formulas are summarized, and the error of each formula for a given gap in its own research is small. However, the errors in other research may become very large. The application of VM has therefore received extensive mass production support. In the calculation conclusions of the theoretical stress concentration factor $K_{t}$ and the notch coefficient $K_{f}$, the relationship between the two is often in line with a linear correlation. The conclusion that can be drawn from the above calculations and the analysis of the graphs is that the polymer PP material theoretical stress concentration factor and the gap factor of the polymer PP material both conform to a linear correlation. The relationship between the coefficient and the gap coefficient is also in line with a linear correlation. More rigorously, it can be said that the two are a positive correlation, and the data obtained in the experimental verification are in line with the theoretical calculation results.

In the calculation of the theoretical stress concentration factor $K_{t}$, the main influencing factors are the initial crack depth and the size of the filling pressure. The value of the pressure is calculated by assuming the initial crack depth, and the value of the pressure can also be calculated by using the measured force on the spot. The size of the notch radius is positively correlated with the theoretical stress concentration coefficient. When the initial crack depth is close to the crack depth, the theoretical stress concentration coefficient $K_{t}$ is also large. The experimental and simulation results verified that the relationship between the theoretical stress concentration coefficient and the notch coefficient of polymer PP material is also in line with a linear correlation, and to be more precise, it can be said that they have a positive correlation.

## Figures and Tables

**Table 1:** Calculation formula of correlation theory stress concentration factor $K_{t}$ (PP).

Notch Radius	0.46 mm		0.51 mm	
a_0_ (mm)	0.33	0.35	0.38	0.40
б	234.973 N	221.546 N	210.112 N	204.653 N
A	108.13 mm^2^		94.25 mm^2^	
F0 = б/A (N/mm^2^)	2.173	2.049	2.229	2.171
F (MPa)	21.402		18.297	
$K_{t}$	9.989	10.445	8.209	8.428

**Table 2:** Parameters of notch radius/$K_{t}$/material constant/$K_{f}$.

Notch Radius	$K_{t}$	Material Constant	$K_{f}$
0.46 mm (a_0_ = 0.33)	10.979	1	3.144
0.46 mm (a_0_ = 0.35)	11.644	1	3.353
0.51 mm (a_0_ = 0.37)	8.209	1	2.374
0.51 mm (a_0_ = 0.39)	8.428	1	2.509

**Table 3:** Corresponding values record of tensile strength of specimen.

Item	Time	Tensile Strength (N)	Deformation Displacement (mm)	Fill Pressure (MPa)
1	12.6167	237.95	2.09263	36.4963
2	12.6333	237.96	2.09543	36.4965
3	12.6528	237.98	2.09823	36.5027
4	12.6667	238.75	2.10103	36.5111
5	12.6833	238.04	2.10377	36.5112
6	12.7654	238.52	2.10652	36.5261
7	12.7167	237.93	2.10931	36.5312
8	12.7333	237.96	2.11206	36.5351
9	12.7529	237.87	2.11486	36.5423
10	12.7667	237.79	2.11766	36.5522

**Table 4:** Different fillet and corresponding values.

	Cross-Sectional Area	Tensile Strength (N)	F0 (N/mm^2^)	$K_{t}$	$K_{f}$
0.46 mm	108.13 mm^2^	238.04N	2.201	13.970	5.402
0.51 mm	94.25 mm^2^	238.04N	2.526	7.243	3.446

**Table 5:** VM and RPM control corresponding values.

	V-P Press	V-P Pos (mm)	Temperature	Cross-Section Stress
	(kg/cm^2^)	(mm)	(Degree)	(MPa)
Average	36.5205	14.0136	243.6225	37.4848
Range	0.3723	0.0234	0.7700	39.9650
SD	0.1715	0.0118	0.3806	17.7108
R/A	0.0102	0.0017	0.0032	1.0662
Coefficient	0.0047	0.0008	0.0016	0.4725
S/N	−31.2508	−22.9310	56.1239	6.5123

**Table 6:** Formula of stress concentration factor $K_{t}$ (polymer PP materials with glass fiber).

Notch Radius	0.46 mm		0.51 mm	
a_0_ (mm)	0.33	0.35	0.38	0.40
б	234.973 N	221.546 N	210.112 N	204.653N
A	108.13 mm^2^		94.25 mm^2^	
F0 = б/A (N/mm^2^)	2.173	2.049	2.229	2.171
F (MPa)	14.494		13.188	
$K_{t}$	6.670	7.074	5.917	6.075

**Table 7:** Parameters of notch radius/$K_{t}$/material constant/$K_{f}$. (PP with glass fiber).

Notch Radius	$K_{t}$	Material Constant	$K_{f}$
0.46 mm (a_0_ = 0.33)	6.670	1	1.786
0.46 mm (a_0_ = 0.35)	7.074	1	1.914
0.51 mm (a_0_ = 0.37)	5.917	1	1.661
0.51 mm (a_0_ = 0.39)	6.075	1	1.714

**Table 8:** Comparison of theoretical $K_{t}$ and $K_{f}$ between PP materials (with glass fiber) and PP materials (no glass fiber).

Crack Depth and Notch Radius	a_0_ = 0.33 mm ρ = 0.46 mm		a_0_ = 0.35 mm ρ = 0.46 mm		a_0_ = 0.37 mm ρ = 0.51 mm		a_0_ = 0.39 mm ρ = 0.51 mm	
Material	PP	PP + GF	PP	PP + GF	PP	PP + GF	PP	PP + GF
$K_{t}$	9.989	6.670	10.445	7.074	8.209	5.917	8.428	6.075
$K_{f}$	2.832	1.786	2.975	1.914	2.374	1.661	2.509	1.714

**Table 9:** Comparison of theoretical warpage and stress between PP materials (no glass fiber) and PP materials (with glass fiber).

	PP (No Glass Fiber)			PP (With Glass Fiber)		
Notch Radius	Warpage (mm)	Direction	F (MPa)	Warpage (mm)	Direction	F (MPa)
0.46 mm	1.3950	Positive	21.402	1.368	Positive	14.494
0.51 mm	1.4318	Positive	18.672	1.288	Positive	13.188

## Data Availability

Not applicable.
